# FRAILTY MEDIATES SENILITY IN MEXICAN AMERICANS

**DOI:** 10.1093/geroni/igz038.1073

**Published:** 2019-11-08

**Authors:** Raymond F Palmer, Donald R Royall, Brian Downer, Kyriakos Markides

**Affiliations:** 1 Department of Family & Community Medicine, The University of Texas Health Science Center at San Antonio, San Antonio, Texas, United States; 2 Departments of Psychiatry, Medicine and Family & Community Medicine, The University of Texas Health Science Center at San Antonio, San Antonio, Texas, United States; 3 University of Texas Medical Branch, Galveston, TX, Galveston, Texas, United States

## Abstract

The latent construct “d” (for “dementia”) offers a continuously distributed transdiagnostic dementia severity metric. Age is significantly associated with “d”. We test whether frailty mediates age’s effect on 6 year prospective change in dementia severity in Mexican-Americans (MA), using data from the Hispanic Established Population for Epidemiological Studies in the Elderly (HEPESE). Age was regressed onto the 6yr prospective slope of change in “d” in N = 880 [mean age = 77.4 (6.1) at wave 3]. Change in “d” was estimated by a latent growth curve (LGC) indicated by latent cognitive measures across three HEPESE waves (i.e., 3, 5 and 6). “Frailty” was assessed by a modified version of Fried et al.’s construct observed at wave 5, and was tested as a mediator of age’s association with change in “d”. The mediation effect was estimated by MacKinnon’s method. “d” at each wave, and the LGC of change in “d” all had acceptable model fit (e.g. RMSEA <.05). Age was significantly associated with change in “d”. 51% of their association was explained by frailty. Frailty mediates the majority of age’s association with dementia severity. Not only does this support the existence of a cognitive “frailty” syndrome in MA, it also implicates an effect of frailty on intelligence (as “d” is derived from Spearman’s general intelligence factor “g”). Their association may be mediated by blood-based serum biomarkers, including somatomedins, which may offer targets for the treatment and /or prevention of senility in frail elderly persons.

